# Achalasia: unusual cause of chronic cough in children

**DOI:** 10.1186/1745-9974-4-6

**Published:** 2008-07-24

**Authors:** Nighat F Mehdi, Miles M Weinberger, Mutasim N Abu-Hasan

**Affiliations:** 1 Pediatrics Pulmonary Section, University of Oklahoma, 940 NE 13th Street, Room 3B 3314, Oklahoma City, OK 73104, USA; 2 Pediatric Allergy and Pulmonary Division, 2544 JCP, University of Iowa, 200 Hawkins Dr, Iowa City, IA 52242, USA

## Abstract

Achalasia is a rare motility disorder of the esophagus which results from lack of enervation of the lower esophageal sphincter muscles and leads to dilatation of proximal esophagus. Patients with achalasia presents typically with dysphagia, vomiting of undigested food and failure to thrive. Cough can be present in achalasia patients due to aspiration of food or due to airway compression by the dilated esophagus. We report two cases of achalasia presenting primarily with prolonged cough. Diagnosis of achalasia in both cases was delayed due to this atypical presentation. This highlights the importance of recognizing achalasia as a potential cause of chronic cough in order to avoid delayed diagnosis and mismanagement.

## Background

Achalasia is a disorder of esophageal motility which occurs rarely in children [1]. Achalasia is caused by loss of inhibitory enervation of lower esophageal sphincter and is characterized by failure of the sphincter to relax. This failure of relaxation causes poor emptying of the esophagus and subsequent dilatation and abnormal contractility of the proximal esophagus. The most commonly presenting symptoms of achalasia include dysphagia, chest pain, vomiting, belching, regurgitation of undigested food and failure to thrive. Cough can occur in achalasia primarily due to aspiration of the undigested food particles or airway compression from dilated esophagus[2].

Due to its rare occurrence, achalasia is not commonly thought of in evaluating children with chronic cough and diagnosis can be consequently delayed. We report two cases of achalasia in children presenting primarily with chronic cough.

## Case 1

A 9-your old girl presented for evaluation of 18 month history of nocturnal cough and post-tussive emesis. Cough occurred mainly at nighttime, occasionally triggered by exercise and was almost always followed with non-bilious vomiting of semi-digested food. Several courses of antibiotics had been given with no improvement in symptoms. Codeine containing cough suppressants were only temporarily effective. There was no response to albuterol inhaler, oral antihistamines and nasal steroids. Besides cough and post-tussive emesis, patient's parents also described less bothersome symptoms of nausea, gagging and epigastric pain. Past medical history was remarkable for being diagnosed with pneumonia a year ago and with bronchiolitis in infancy.

On examination, she was above the 25^th ^percentile for weight and above the 75^th ^percentile for height. Vital signs were normal. Chest exam showed no signs of respiratory distress and was clear to auscultation. Initial evaluation showed normal chest x-ray, normal lung spirometry, normal exercise challenge, and negative skin testing to common inhaled allergens.

Combined endoscopy and bronchoscopy were done under conscious sedation. Endoscopy identified no abnormality of the esophagus or stomach. Bronchoscopy, on the other hand, showed oval shaped trachea at its mid portion about 2.5 cm above the carina. The anterior and posterior walls of the trachea approach each other, especially during vigorous coughing, and came to complete contact on the right side creating a teardrop shaped lumen. No abnormalities of the bronchi were observed. Broncoalveolar lavage (BAL) fluid cell count contained 8% lymphocytes, 7% neutrophils, 10% eosinophils and 70% macrophages. BAL culture grew only mixed flora.

**Figure 1:**
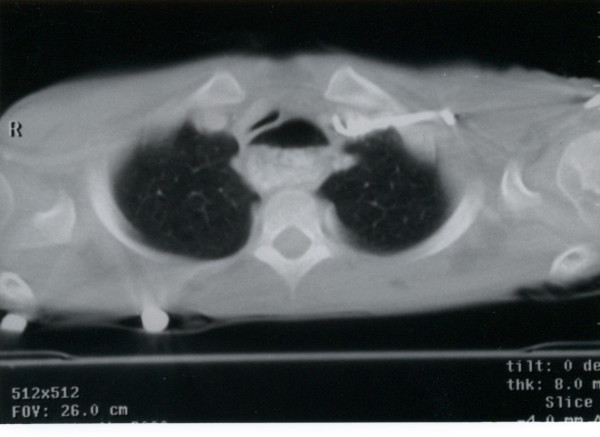
**CT scan of chest showing dilatation of esophagus with air-fluid level.** Dilated esophagus is compressing the trachea anteriorly.

**Figure 2:**
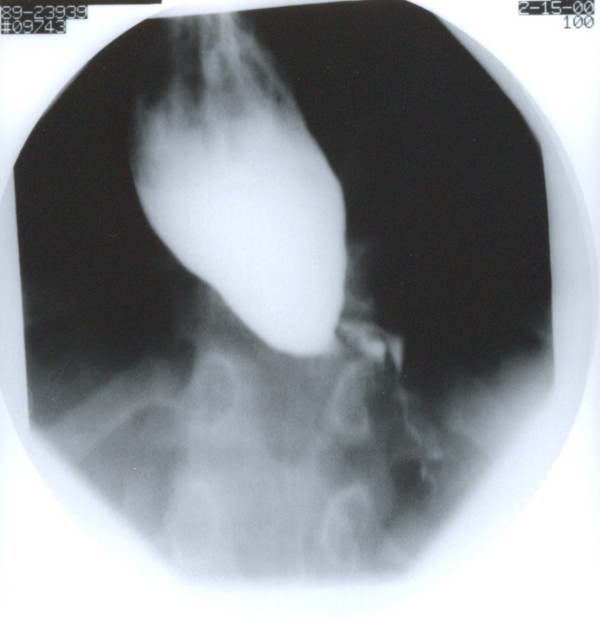
Esophagogram showing severe dilatation of esophagus with smooth tapering at the gastroesophageal junction.

Because of the mid tracheal collapse observed during bronchoscopy, a cine-CT with contrast was done to rule out vascular ring with compression of the trachea. The CT showed no abnormal vasculature but did show a large dilated esophagus with air-fluid level from stagnant food material (Figure 1). The trachea was compressed by the dilated esophagus and deviated towards the right. A Barium swallow study done later confirmed the diagnosis of achalasia and demonstrated the presence of megaesophagus with tapering and marked narrowing at the gastroesophageal junction causing functional obstruction with significant delay of contrast passage into the stomach (figure 2). There were no primary peristalsis throughout most of the esophagus and only non-peristaltic contractions were seen. No aspiration was observed. Patient underwent corrective surgical procedure after which the symptoms of chronic cough disappeared.

## Case 2

A 10-year old African-American female with Down syndrome was evaluated by our pulmonary service for history of chronic daily cough and recurrent pneumonias for eight and a half years duration. Cough was worse at night, in supine position and during exertion. Cough also worsened during viral respiratory tract infections. There was history of frequent vomiting of undigested food but not necessarily associated with the cough.

Patient was diagnosed with asthma exacerbations and pneumonia and treated as such several times in her lifetime. She had history of transient clinical improvement with antibiotics, bronchodilators and oral corticosteroids. Daily inhaled corticosteroids failed to completely control cough. Past medical history was significant for small ventricular septal defect and chronic constipation.

On examination, she was between 75^th ^and 90^th ^percentile for weight, and between 10^th ^and 25^th ^percentile for height. Chest examination was unremarkable. Chest roentgenograms showed predominantly patchy peribronchial air space consolidation with more involvement of the right middle lobe. A Barium swallow study was first done at 5 years of age. There was no frank aspiration or laryngeal penetration and the esophagogram revealed normal anatomy of the esophagus with mildly delayed hypopharyngeal contraction but no contrast retention.

Flexible bronchoscopy showed normal airway anatomy. The BAL fluid contained 16% neutrophils, 35% lymphocytes, 35% macrophages with 14% percent epithelial cells. Cultures grew only mixed flora. Lipid laden macrophages were present with Colombo index of 110 which is consistent with aspiration[3].

**Figure 3:**
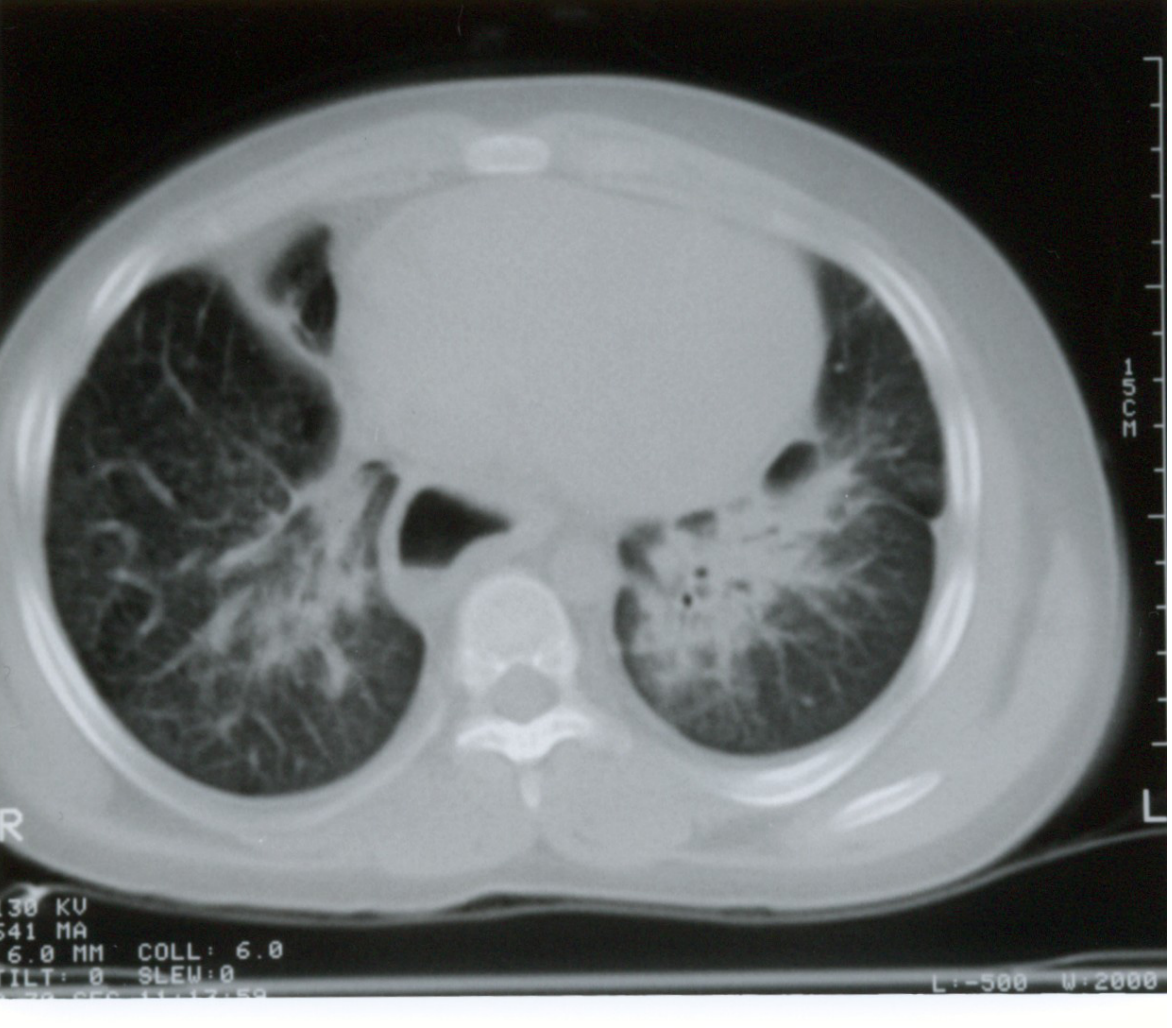
CT scan of chest showing bilateral lung opacification with septal thickening and esophageal dilatation.

**Figure 4:**
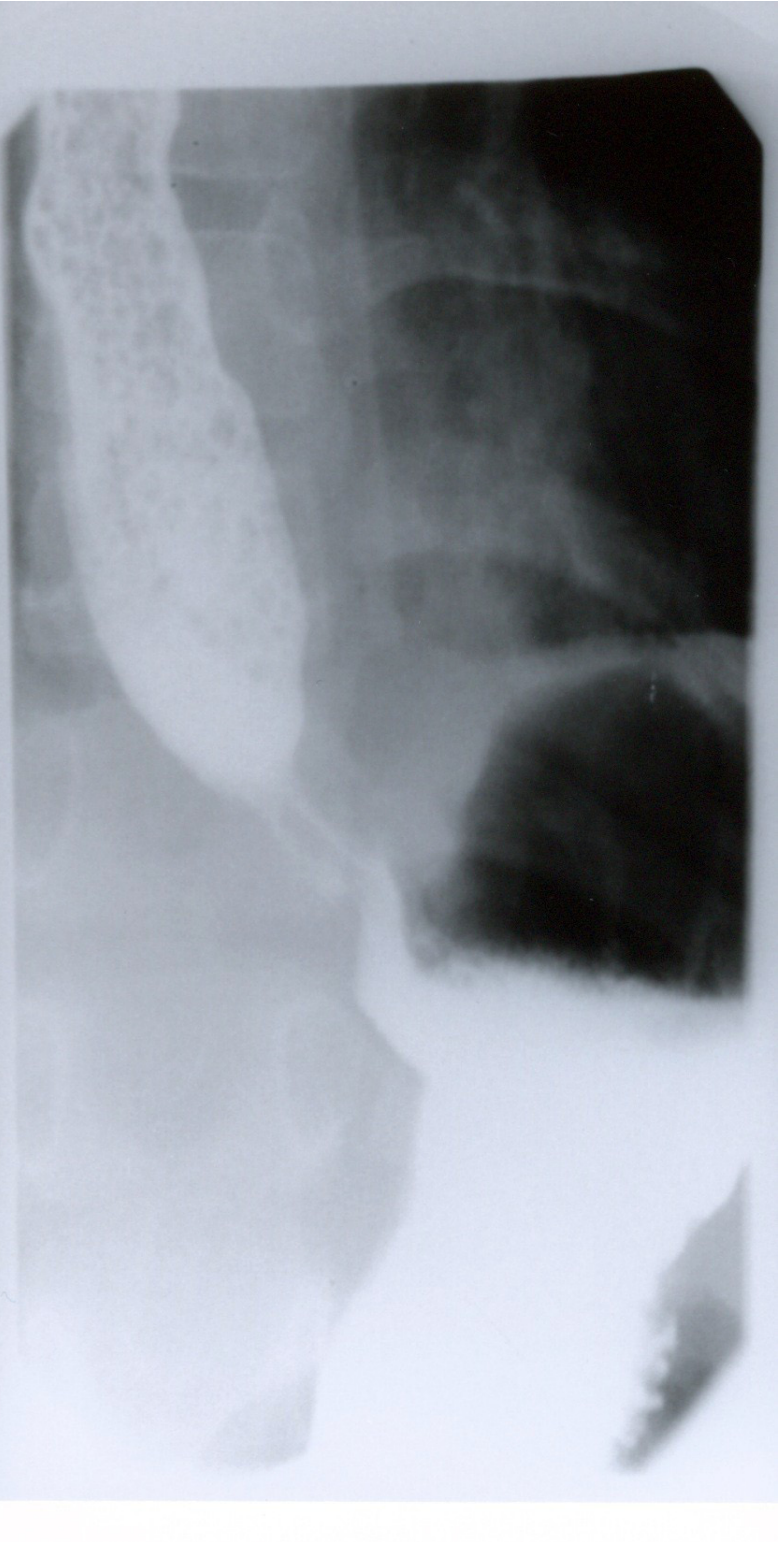
**Esophagogram showing diffuse dilatation of esophagus with tapering at gastroesophageal junction.** Esophagus appears filled with food particles.

CT scan of the chest showed diffuse lung infiltrates that seemed worse on right middle lobe and left lower lobe. The esophagus was enlarged along its entire length but with no obvious tracheal compression (figure 3). In view of the CT scan findings, a repeat Barium swallow study was done which showed severe esophageal dysfunction with multiple waves of tertiary contractions throughout a markedly dilated thoracic esophagus. the distal esophagus appeared tapered caudally with severe narrowing at the gartroesophageal junction. Small food particles with air bubbles were seen within the mid esophagus. Only small amount of contrast passed to the stomach (figure 4).

## Discussion

Achalasia is the most commonly recognized esophageal motor disorder, first described 300 years ago by Sir Thomas Willis[4,5]. It is an idiopathic esophageal motility disorder, characterized by absence of normal peristalsis and failure of relaxation of lower esophageal sphincter. Achalasia occurs in the general population with a prevalence of eight per 100,000. It occurs mainly in adulthood, with less than 5% of cases found before adolescence[1]. It has been associated with other diseases such as Parkinson's disease, progressive cerebellar ataxia, familial glucocorticoid deficiency and Down syndrome[6,7]. Childhood achalasia is more common in boys[8]. Few cases have been reported in infants [9]. Familial forms are rare.

The most frequent symptoms of achalasia are dysphagia, chest pain, regurgitation of food, and weight loss. Secondary pulmonary disease can occur due to regurgitation and aspiration of retained esophageal contents. This can cause symptoms of chronic cough, especially nocturnal cough, choking, recurrent pulmonary infections, pneumonia, wheezing, atelectasis and rarely pulmonary empyema [10]. Some patients may develop hoarseness of the voice caused by direct pressure of distended esophagus on the recurrent laryngeal nerve [11].

Tracheal obstruction due to compression from dilated esophagus may occur in achalasia and can be the only presentation [12]. This can be a serious and potentially life threatening complication of achalasia [13,14].

Diagnosis of achalasia is suspected by the presence of dilated esophagus with tapering of distal esophagus on contrast esophagography (bird peak appearance). Esophagography, however, suffers from low sensitivity as a diagnostic test [15]. CT scan of chest can also detect dilated esophagus, esophageal wall thickening, and stagnant food [16]. CT scan is particularity helpful in detecting associated comorbidities such as malignancies and lung infiltrates [17]. The diagnostic value of chest CT for achalasia compared to manometry has not been systematically studied. Pressure manometry detects motility dysfunction of the esophagus and failure of relaxation of the distal esophageal sphincter and is considered the golden standard for diagnosis of achalsia. If biopsy of distal esophagus is done, lack of myenteric plexus enervation can be demonstrated.

In this report both cases presented with chronic nocturnal cough as the main presenting symptoms of achalasia. Other symptoms such as vomiting of undigested or semi digested food was present but were not enough red-herring to raise suspicion for diagnosis. Diagnosis of achalasia was finally reached after prolonged history of symptoms and after several non-diagnostic evaluations and several empirical therapies. Interestingly, diagnosis in both cases was made by CT scan finding of dilated esophagus and then confirmed by esophagography showing the dilated, tapering and dysfunctional esophagus. Even though, an esophagogram was done earlier in the second case, the diagnosis of achalasia was not made on that study. Meanwhile, patient continued to have cough and recurrent aspiration pneumonia. The severely dilated esophagus was seen years later on the CT scan and became more obvious in the second esophagogram.

The second case had more severe complications from aspiration than the first case despite similar age of presentation. This could be due to poor airway clearance and the non-specific immune compromise associated with trisomy 21. The presence of esinophilia in the brochoalveolar lavage could not be clearly explained. It could possibly reflect an associated atopic airway inflammation or asthma.

The presence of trachea compression on the CT scan in the first case suggests that tracheal compression was the mechanism of chronic cough. On the other hand, the presence of extensive pulmonary infiltrates on the chest CT scan and the high lipid laden macrophage index in the BAL fluid suggests that chronic aspiration was the mechanism of chronic cough in the second case [[18,19],20].

In conclusion, both cases clearly demonstrate that achalasia could present primarily as chronic cough due to tracheal compression from dilated esophagus and/or chronic aspiration from regurgitated food. They also demonstrate that failure to entertain the likelihood of achalasia as a cause of chronic cough could result in late diagnoses and unwarranted morbidity.

## Consent 

The patients are lost to our follow-up, so obtaining consent is not possible.  Verbal consent was obtained by the treating physicians and the coauthors (Dr. Mehdi and Dr. Weinberger)

## Authors' contributions

All authors conceived of the study, and participated in its design and coordination. All authors read and approved the final manuscript
